# Precision diagnosis and treatment of vitamin metabolism-related epilepsy

**DOI:** 10.1186/s42494-024-00169-0

**Published:** 2024-10-01

**Authors:** Yajing Gan, Guoyan Li, Zihan Wei, Yan Feng, Yuqing Shi, Yanchun Deng

**Affiliations:** 1 Department of Neurology, Epilepsy Center of Xijing Hospital, Fourth Military Medical University, Xi’an, 710032 People’s Republic of China; 2 Xi’an Medical University, Xi’an, 710021 People’s Republic of China; 3 Xijing Institute of Epilepsy and Encephalopathy, Xi’an, 710000 People’s Republic of China

**Keywords:** Epilepsy, Metabolism, Vitamin, Treatment

## Abstract

Epilepsy is a chronic disorder of the nervous system caused by abnormal discharges from brain cells. Structural, infectious, metabolic, immunologic, and unknown causes can contribute to the development of seizures. In recent years, there has been increasing attention on epilepsy caused by genetic metabolic disorders. More than two hundred inherited metabolic disorders have been identified as potential cause of seizures, and they are mainly associated with energy deficiency in the brain, accumulation of toxic substances, abnormal neurotransmitter transmission, and deficiency of cofactors. Vitamins play a crucial role as components of several enzymes or coenzymes. Impaired metabolism of thiamine, biotin, vitamin B6, vitamin B12 and folic acid can contribute to early-onset seizures and developmental abnormalities in infants. However, timely supplementation therapy can significantly improve patient prognosis of affected patients. Therefore, a thorough understanding and investigation of the metabolic basis of epilepsy is essential for the development of precise therapeutic approaches, which could provide significant therapeutic benefits for patients.

## Background

Epilepsy is a recurrent and transient disorder of the central nervous system characterized by excessive discharge of nerve impulses in the brain [1]. Previous seizure-related studies have focused on changes in synaptic transmission. However, as research progresses, disturbances in cellular and mitochondrial metabolism have been recognized as significant contributors to the development of epilepsy. Disorders in brain metabolism and seizures may interact, exacerbating the condition. The current global prevalence of inborn errors of metabolism is approximately 1 in 2000 people, with 20–30% of individuals experiencing epilepsy as their primary neurological manifestation [2]. Oral antiseizure medication therapy is currently the routine treatment for patients with epilepsy, although standardized and appropriate antiseizure medications can control seizures in 70–80% of cases. However, nearly 30% of patients eventually develop drug-refractory epilepsy, which significantly affects the quality of life and psychological health of patients, posing a major societal burden [1]. Among them, mutation-associated epilepsy, in particular, is more likely to progress to drug-resistant epilepsy. Over eight hundred epilepsy-related genes have been identified, with about 42% of them being metabolically related [3]. Therefore, understanding and studying the metabolic basis of epilepsy will facilitate the development of more innovative diagnostic and treatment strategies with greater therapeutic benefits.

Currently, genetic metabolic diseases cause epilepsy mainly through mechanisms such as energy deficiency, toxic effects, disturbance of the neurotransmitter system, and cofactor deficiency. Among them, vitamins, which serve as coenzymes or coenzymatic components of a variety of enzymes, are not involved in the composition of body tissues and cells or in the production of energy. However, they are mainly responsible for regulating the body's metabolism, producing energy, building and transforming biologically active molecules, and playing an important role in maintaining the function of the nervous system. Vitamin deficiencies can therefore lead to a number of systemic metabolic disorders, collectively referred as vitamin deficiencies. Deficiency in any of the B vitamins can result in neuropsychiatric symptoms. Specifically, disorders in the metabolism of vitamin B6, thiamine, biotin, vitamin B12, and folic acid metabolism disorders can contribute to drug-resistant seizures and developmental abnormalities in early infancy. Nevertheless, timely treatment with appropriate vitamin supplementation early in life can effectively control seizures and even restore patients to normalcy. Therefore, this review provides a summary of the clinical features and precise treatment protocols for epilepsy related to vitamin metabolism. The aim is to enable clinicians to identify such disorders at an early stage and improve the prognosis of patients.

## Main text

### Vitamin B6 metabolism disorders and epilepsy

Vitamin B6 encompasses six compounds, namely pyridoxine (PN), pyridoxal (PL), and pyridoxamine (PM), along with their respective 5-phosphates. Of these, pyridoxal 5'-phosphate (PLP) is the most metabolically active form and acts as an enzyme cofactor in the biochemical metabolism of amino acids. Vitamin B6-dependent epilepsy is a group of autosomal recessive disorders caused by mutations in the genes for phosphatidylinositol binding protein (PLPBP), acetaldehyde dehydrogenase 4 family member A1 (ALDH4A1), acetaldehyde dehydrogenase 7 family member A1 (ALDH7A1), pyridoxal (amine) 5'-phosphate oxidase (PNPO), and tissue non-specific alkaline phosphatase (TNSALP).

In the human body, vitamin B6 is primarily absorbed in the small intestine, where it enters the portal vein and subsequently reaches the liver, then it is converted into PL, PN and PM as well as their respective 5'-phosphates [4]. A portion of pyridoxine 5'-phosphate (PNP) and pyridoxamine 5'-phosphate(PMP) is oxidized by PNPO to PLP, which is bound to albumin in the blood and travels to all parts of the body. In the central nervous system, PLP is converted to PL across the blood-brain barrier in the presence of TNSALP. Inside brain cells, PL is phosphorylated back to PLP. PLP acts as a universal catalyst and cofactor for amino acids or amines. In the brain, many PLP-dependent enzymes are involved in the metabolism of crucial amino acid or amine neurotransmitters such as dopamine, GABA, glycine, glutamate, and 5-hydroxytryptamine. Therefore, PLP dysfunction can lead to serious central nervous system disorders such as seizures [5].

#### *PLPBP* gene mutation and epilepsy

The *PLPBP* gene, located on chromosome 8p11.23, encodes pyridoxal phosphate homeostasis protein (PLPHP), which is widely expressed in humans, and can be found in the mitochondria or cytoplasm. Its role is to bind PLP and ensure its availability for coenzymes, thus avoiding PLP reacting with other substrates or being phosphorylated by intracellular phosphatases [4]. Therefore, mutations in the *PLPBP* gene disrupt this process, causing PLP to react with other substrates and impacting the metabolism of important amine or amino acid neurotransmitters in the brain, consequently leading to the development of epilepsy. To date, a total of 54 patients with mutations in the *PLPBP* gene have been reported, with approximately 63% of them have consanguineous parents [6]. Among these cases, 14 patients carried the c.370_373delGACA mutation, which belongs to the Saguenay-Lac-St-Jean French Canadian founder mutation, in homozygosity and/or compound heterozygosity state. Patients with homozygosity at this mutated locus tend to exhibit a more severe clinical phenotype, usually associated with severe mental retardation and early onset seizures, and five of them died early in life [6–8]. Pregnancy complications, such as preterm birth, fetal distress, and intrauterine hypermobility, were observed in approximately 34% of patients. Almost all preterm infants develop seizures within the first 24 hours of life, while other affected infants usually develop seizures within the first week of life. Generalized tonic-clonic (GTC) is the most common seizure type, followed by tonic, clonic, myoclonic, seizures with impaired awareness (SIA), and spasms. In addition to seizures, infantile anemia, infantile gastrointestinal dysfunction, necrotizing small bowel colitis, gastroesophageal reflux disease, and metabolic acidosis may occur [4]. Developmental delay, mental retardation, autism spectrum disorder, acquired microcephaly, and psychiatric abnormalities are also commonly observed in most patients [4, 6, 9, 10]. Cranial MRI may reveal simplified gyri, structural brain abnormalities, periventricular cysts, enlarged ventricles, thinning of the corpus callosum, and high signal in the white matter of the brain.

In individuals with *PLPBP* mutations, the most common metabolic abnormality is an increase in blood lactate and glucose levels, leading to metabolic acidosis shortly after birth, typically on day 1, then returns to normal by day 3 to 4 [11]. Plasma amino acid metabolism is most commonly associated with elevated levels of glycine [11]. CSF examination also shows elevated lactate levels in addition to low homovanillic acid (HVA), along with high levels of 3-O-methyldopa, levodopa, and 5-hydroxytryptophan, suggesting the dysfunction of aromatic L-amino acid decarboxylase (AADC). A study by Akiyama et al. found that reduced CSF PLP concentrations may be a more sensitive indicator of vitamin B6-dependent epilepsy, especially vitamin B6-dependent epilepsy caused by mutations in *PLPBP* [12]. However, it is important to acknowledge that *PLPBP* still lacks specific diagnostic features and markers for early detection and prognostic assessment [11]. Therefore, the diagnosis of PLPHP deficiency still relies on standard B6 testing and genetic testing. It is crucial to conduct standard vitamin B6 testing in an environment with full resuscitation capabilities [13].

Patients with vitamin B6 deficiency typically show insensitivity to antiseizure medications but respond well to treatment with PN, and there is a clear dose-dependent relationship [6]. Nearly 91% of patients with *PLPBP* mutations responded to initial treatment with PN and PLP, and about 82% and 50% of patients achieved seizure freedom with PN and PLP, respectively [8]. Long-term maintenance treatment achieves seizure freedom in 69% of patients, with PN in combination with antiseizure medications having the best efficacy. Therefore, it is crucial to start PN treatment as soon as patients are diagnosed with *PLPBP* gene mutations [14]. Switching to PLP may be considered in patients who do not respond well to PN treatment. According to guideline, it is advised that the daily dose of PN should not exceed 200–300 mg in order to prevent PN overdose, which can lead to nerve damage and motor dysfunction [13, 15]. Vitamin-dependent epilepsy requires continuous drug maintenance therapy, and symptoms may return when the drug is discontinued [14]. For patients under 3 years of age with unexplained seizures, it is recommended to undergo intravenous PN testing before starting regular antiseizure medication. Lifelong PN supplementation is initiated if the PN test is positive, and if it is negative, oral PN is continued for several days to avoid overlooking delayed responses.

#### *ALDH4A1* gene mutation and epilepsy

**Fig. 1:**
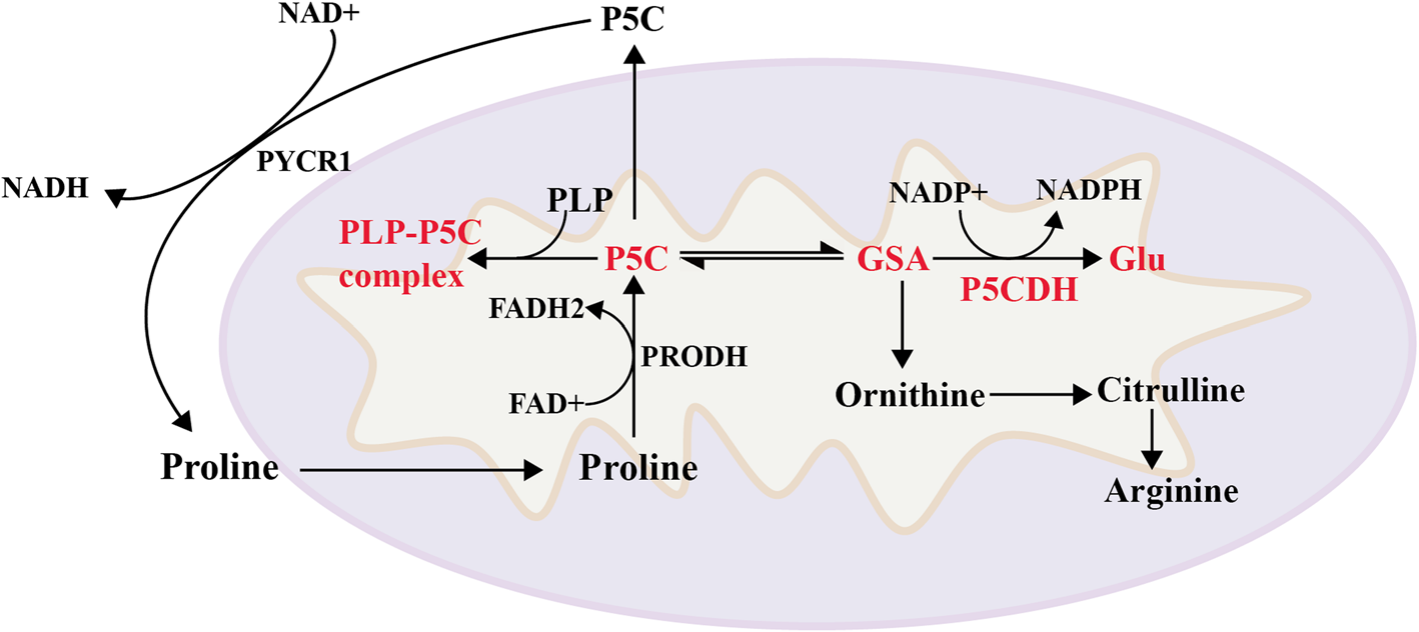
*ALDH4A1* mutation leads to accumulation of P5C in mitochondria. P5C reacts with PLP to form PLP-P5C condensate, which reduces the concentration of PLP in the brain. Elevated P5C can be converted to proline, leading to mitochondrial stress

The *ALDH4A1* gene, located at 1p36.13, encodes delta(1)-pyrroline-5-carboxylate dehydrogenase (P5CDH). Mutations in this gene can cause type II hyperprolinemia (HPII) with a estimated prevalence of approximately 1 in 700,000 [16]. The mutation in *ALDH4A1* leads to the accumulation of pyrroline-5-carboxylate (P5C) in the mitochondria, which can react with PLP to cause PLP deficiency in the brain and can also be converted to proline, resulting in elevated plasma proline levels (Fig. 1) [17]. In patients with hyperprolinemia, proline accumulation can cause a decrease in ATPase activity, which further leads to mitochondrial stress and elevated lactate levels, and can also decrease glutamate uptake [17]. Thus increased oxidative stress, loss of Na^+^/K^+^-ATPase activity, and altered biochemical markers of mitochondrial function play a role in the increased excitotoxicity in individuals with hyperprolinemia.

The most common clinical manifestations are recurrent seizures associated with fever, usually occurring in the neonatal period, early infancy or early childhood. Additionally, individuals may experience a range of developmental delays, mental retardation and significant behavioral problems, including schizophrenia, anxiety and hallucinations. Systemic symptoms such as upper respiratory tract infections, vomiting, persistent diarrhea and abdominal pain, respiratory failure, hyperlactatemia and hyperkalemia can also be present [16, 17]. However, mild hyperprolinemia is not associated with seizures. So far, a total of eight different types of *ALDH4A1* variants have been reported, and Kaur et al. reported the first case of a nonsense mutation leading to premature translation termination [18].

Lactic acidosis must usually be ruled out before HPII is diagnosed, as it can lead to secondary hyperprolinemia [19]. Diagnosis of HPII can be made based on plasma proline levels, as well as elevated urinary P5C levels, proline, hydroxyproline, and glycine, which serve as important markers. HPII can be differentiated from HPI on the basis of urinary P5C levels, but the gold standard for diagnosis is still genetic testing [19].

Although seizures in patients with *ALDH4A1* mutations may respond to treatment with PN or PLP, these interventions do not improve their intellectual development. According to Hassel et al., the accumulation of γ-glutamate semialdehyde (GSA) and P5C in the brain may contribute to the development of mental retardation in patients [20]. It has been demonstrated that in animals with *ALDH4A1* mutation, vitamin E and vitamin C supplementation can reverse the inhibition of ATPase activity by hyperprolinemia, and appropriate use of antioxidants can also be helpful to reduce neurotoxicity [19].

#### *ALDH7A1* gene mutation and epilepsy

**Fig. 2:**
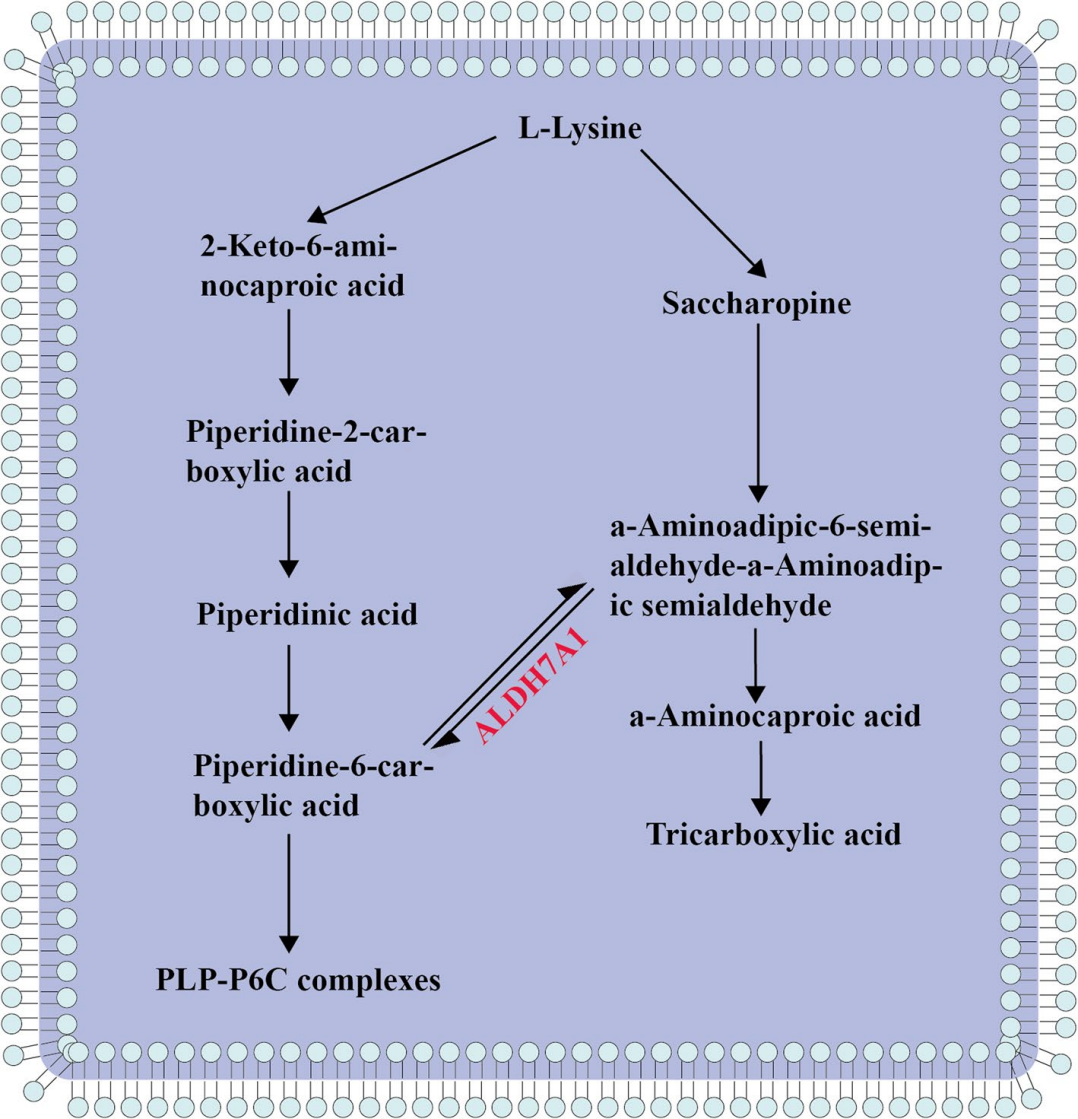
Lysine metabolism. *ALDH7A1* is involved in the oxidation of lysine and mutations in this gene lead to pathological accumulation of α-AASA, P6C and piperonylic acid. P6C accumulation can react with PLP to form a Knoevenagel condensate, which inactivates PLP and interferes with the metabolism of important neurotransmitters in the brain

The *ALDH7A1* gene, located on chromosome 5q23.2, encodes antiquitin, which is responsible for catalyzing the dehydrogenation of α-aminoadipic semialdehyde (α-AASA)/piperideine 6-carboxylate (P6C). Antiquitin is involved in the oxidation of lysine, and deficiency of this enzyme leads to pathological accumulation of α-AASA, P6C, and piperidic acid. In contrast, the accumulation of P6C can react with PLP to form a Knoevenagel condensate, which inactivates PLP and interferes with the metabolism of important neurotransmitters in the brain (Fig. 2) [21]. In particular, it has been suggested that *ALDH7A1* is expressed in glial cells and is involved in early neurogenesis and migration, so mutations in this gene may be associated with abnormal neuronal migration and structural brain disorders [22]. A total of 165 *ALDH7A1* pathogenic variants have been reported in the literature, but no clear genotype-phenotype correlation has been established. The estimated prevalence of the disease in healthy populations is 1 in 64,352, with a prevalence of 1 in 16,556 in Asia. The most common mutated loci reported in the literature are c.1279G>C and p.Glu427Gln, with 98% of patients having a double allele pathogenic variant [23, 24].

Patients with *ALDH7A1* mutations may develop abnormal fetal movements or even intrauterine distress during fetal life. The typical form of seizures occurs within the first month of life, while in atypical patients, seizures may be delayed until infancy to 3 years of age. Seizures can manifest in a variety of forms, but generalized tonic-clonic seizures (GTCS) predominate. Fever can act as a trigger for some patients. In addition to seizures, patients may experience other neuropsychiatric symptoms including autism spectrum disorder, anxiety, attention-deficit/hyperactivity disorder (ADHD), obsessive-compulsive disorder, mood disorders, hypotonia or hyperactivity, hypervigilance, and sleep disorders. Although treatment with PN may provide partial response, 75% of patients still exhibit residual developmental delays and mental retardation [25]. Systemic symptoms such as vomiting, poor feeding, strabismus, macrocephaly, hypotension, respiratory distress, microcephaly, thrombosis, *E. coli* sepsis, cataracts, hepatomegaly, coagulopathy, uropathy, necrotizing small bowel colitis, jaundice, and hypothermia are less common [25]. Severe metabolic and endocrine disturbances such as hypocalcemia, hypomagnesemia, hypoglycemia, hypothyroidism, and uroparesis, can also occur in a small number of patients [24, 26].

MRI may show structural brain abnormalities such as enlarged ventricles, hypoplasia of the corpus callosum, cortical atrophy, myelin hypoplasia and congenital hydrocephalus, which may be associated with impaired neuronal migration. Brainstem and pontine dysplasia are rare phenotypes, with less than five patients reported [25]. The most common discharge pattern on the EEG is burst suppression, followed by high amplitude dysrhythmias and multi-spike discharges, with corresponding changes in the EEG after the patient receives PN treatment [27]. Plasma threonine, glycine, taurine, histidine, and 3-methoxytyrosine levels are increased in 50% of patients with *ALDH7A1* mutation. CSF levels of GABA, glutamate, threonine, glycine, taurine, histidine, and 3-methoxytyrosine were decreased. Mills et al. analyzed 272 samples and found a significantly higher α-AASA/creatinine ratio in this group of patients with a significant age dependency [24]. Urine α-AASA levels should be measured in all newborns with refractory seizures. A significant correlation was found between 6R-oxopropylpiperidine-2-carboxylic acid (2-OPP) and *ALDPH7A1* gene deficiency in patients. Urinary levels of 2-OPP showed a significant positive correlation with α-AASA, and the highest levels of 2-OPP in the CSF were observed in patients treated with vitamin B6. Animal studies suggest that excessive levels of 2-OPP in the brain may lead to persistent neurotoxicity and induce seizures [28].

Patients with *ALDH4A1* gene mutations typically show poor response to antiseizure medications, while vitamin B6 supplementation has been found to alleviate seizures. However, the response to vitamin B6 may be slower in patients whose epilepsy onset occurs later [29]. Although vitamin therapy can be effective, there may still be a legacy of developmental delays and mental retardation [30]. Karnebeek et al. discovered that restricting dietary intake of lysine in patients was well tolerated and resulted in a substantial reduction in plasma and CSF levels of α-AASA and P6C, resulting in seizure control and some improvement in developmental milestones [25]. Furthermore, arginine supplementation was found to reduce CSF α-AASA levels and improve motor-verbal function in patients [31]. Because arginine competitively inhibits lysine transport into the CNS, it reduces the accumulation of P6C and α-AASA [32]. Gajlagher et al. found that the *ALDH7A1* mutation is also present in folinic acid-responsive epilepsy, and combined treatment with PN and folinic acid may be considered for patients who do not respond to PN or folinic acid alone [33]. Overall, the main treatment strategy for this group of patients involves vitamin B6 and arginine supplementation to reduce levels of P6C and α-AASA in the brain. Early prevention is also important, and active screening of high-risk individuals and initiation of treatment prenatally may be effective in preventing brain malformations in affected children.

#### *PNPO* gene mutations and epilepsy

The *PNPO* gene is situated on chromosome 17q21.32 and encodes pyridoxine-5'-prime-phosphate oxidase, a key enzyme involved in the conversion of PN/PM to PLP. Mutations in this gene can lead to reduced levels of PLP, which can affect the metabolism of several essential amino acids or amine neurotransmitters in the brain [34].

Mills et al. found that mutations in the *PNPO* genes R225H/C, D33V, and R116Q/P significantly reduced *PNPO* activity and increased the likelihood of PN-responsive epilepsy [35]. Individuals with R116Q mutations in the *PNPO* gene have a milder phenotype, with some experiencing no seizures or late-onset symptoms and only mild to moderate mental retardation [36]. Additionally, most patients with *PNPO* mutations are born prematurely and exhibit abnormal fetal movements, intrauterine distress, and amniotic fluid abnormalities in the fetus. Seizures usually manifest within the first 2 weeks of life, with a small number of cases may develop after the first year. The epileptic phenotype is diverse, with a predominance of generalized myoclonic seizures, followed by abnormal eye movements and absence seizures to a lesser extent [14]. It is worth noting that multisystem abnormalities can also occur, with anemia and coagulopathy being the most frequent hematologic manifestations. Abdominal distension, feeding difficulties, constipation, hepatomegaly, and other gastrointestinal manifestations may also be present [14]. Approximately 56% of patients have developmental delay and mental retardation. The prognosis is generally poor for those who are born prematurely, have early-onset seizures, and experience delayed treatment [37]. The EEG shows mainly a burst suppression pattern and completely normal patients are rare [14]. The most commonly observed finding on MRI in patients with *PNPO* gene mutations is cerebral atrophy, followed by basal ganglia ischemia, cerebral myelin dysplasia, cortical laminar necrosis and cortical edema. Less commonly, MRI may show intracranial venous sinus thrombosis and subdural hemorrhage [14]. In approximately 80% of patients, elevated levels of glycine can be detected in the CSF, while CSF levels of PLP are decreased in 81% of cases and urinary vanilloid acid is elevated in 91% of patients [37]. Elevated CSF and plasma PM levels and dried blood spot assays showing reduced PNPO enzyme activity are hallmarks of *PNPO* gene mutations [14].

Vitamin B6 therapy should be started as early as possible in this group of patients, as prompt and timely treatment is strongly associated with a favorable prognosis. Patients who do not receive timely treatment usually have severe developmental delays and severe brain damage. The activity of PNPO is essential for the synthesis of PLP, which is the only theoretically effective therapeutic agent, and must be taken every 6 hours. However, 44% of patients only respond to PN, probably due to residual enzyme activity that converts PN to PLP [37]. It is noteworthy that high concentrations of PLP can regulate intracellular PLP levels by inhibiting the activity of PNPO enzyme, whereas mutant enzymes with residual activity cannot be regulated by PLP negative feedback. As a result, some patients experience worsening symptoms after switching from PN to PLP [35]. Long-term administration of high doses of PLP can potentially impair liver function, leading to hepatomegaly, cirrhosis, and, in rare cases, hepatocellular carcinoma. Therefore, patients' liver function should be closely monitored, and patients who clearly respond to PN should continue with PN therapy, and the addition of the PNPO cofactor riboflavin may improve the activity of residual PNPO [14].

#### *TNSALP* gene mutations and epilepsy

The *TNSALP* gene, located on chromosome 1p36.12, encodes a tissue non-specific alkaline phosphatase enzyme, which is widely distributed in the liver, bones, kidneys, and developing teeth. Mutations in the *TNSALP* gene can result in either a complete loss or only 40% of normal alkaline phosphatase activity. However, the degree of reduction in alkaline phosphatase activity is not related to the severity of clinical manifestations [31]. According to the literature, the autosomal recessive inheritance pattern is generally associated with a more severe clinical phenotype compared to autosomal dominant cases [38]. In 1948, Rathbun first described the disease as hypophosphatasia in an infant, presented with severe bone loss, seizures, and low levels of alkaline phosphatase tissue and blood, who died of tonic epilepsy at 2 months of age [39]. The incidence of the disease is approximately 1 in 100,000, and the mortality rate for infantile hypophosphatasia is as high as 50%. In the United States, Whites have shown to be more susceptible to the condition compared to Blacks, and the mortality rate of the disease can further increase when it is associated with infectious atypical pneumonia [40–42]. *TNSALP* provides inorganic phosphate for the production of hydroxyapatite crystals and promotes the excretion of inorganic pyrophosphate (PPI) [43–45]. The growth of these hydroxyapatite crystals eventually breaks matrix vesicles and mineralizes bone-like material [46]. In contrast, PPI disrupts the growth of hydroxyapatite crystals and inhibits bone mineralization. Therefore, mutations in the *TNSALP* gene result in a decrease in hydroxyapatite production, leading to impaired bone mineralization and decreased bone mass [41]. In addition, PLP, a cofactor for more than 140 enzymes, is converted to PL by TNSALP across the blood-brain barrier and then converted back to PLP. When the *TNSALP* gene is mutated, it results in low levels of PLP in the CNS, which leads to PN-responsive epilepsy [47, 48].

Based on previous reports in the literature, hypophosphatemia can be classified into six different forms: adult hypophosphatemia, hypophosphatemia in children, infant hypophosphatemia, perinatal hypophosphatemia, benign prenatal hypophosphatemia, and dental phosphate deficiency. Among these forms, perinatal hypophosphatemia is the most severe, leading to skeletal damage in the fetus that may not apparent during pregnancy, and often resulting in death shortly after birth. Infantile and perinatal hypophosphatemia are also associated with a variety of acute and chronic systemic manifestations, including poor feeding, anorexia, irritability, persistent vomiting, mild anemia and dehydration, growth retardation, scleral opacities, skin pigmentation, cyanosis, pulmonary infections, hypotony, and recurrent fractures. Skeletal deformities include cheekbone enlargement, head circumference reduction, cranial stenosis, limb shortening, knee valgus, and, in very severe cases, spherical skulls. Dental abnormalities such as premature loss of primary teeth, hypoplastic teeth, inadequate alveolar bone growth, enlarged pulp spaces, and severe dental caries may also occur. In some cases, hyperphosphatemia and hypercalcemia may even occur due to blocked mineral entry into the bones. Due to abnormal intracranial PLP transport in patients with *TNSALP* mutations, there is insufficient neurotransmitter synthesis in the brain, which eventually contributes to PL-dependent seizures, usually preceding the onset of skeletal changes [41, 49–52].

In general, an earlier age of onset of bone disease is associated with a poorer prognosis [41]. In addition, elevated serum PLP is a sensitive marker for hypophosphatasia, and serum and urine phosphatidylethanolamine levels are usually elevated. Skeletal radiographs are also helpful in diagnosing the disease. Calcitonin and chlorothiazide may be used to treat patients with hypercalcemia and high urinary calcium [53]. Enzyme replacement therapy for hypophosphatasia has shown promising results in human clinical trials, reducing mortality in hypophosphatasia and improving chondromalacia, lung function and exercise milestones in these patients [44]. Discontinuing PL supplementation after initiating enzyme replacement therapy can prevent epilepsy recurrence [54]. Additionally, bone marrow and bone marrow cell transplantation enhances the activity of TNSALP in the bones, and the therapy is currently working well in two female infants as reported [49, 55].

### Thiamine metabolism disorders and epilepsy

The *SLC19A3* gene is situated at 2q36.3 and encodes thiamine transporter protein 2 (THTR2). *SLC19A1* encodes a reduced folate transporter protein, while *SLC19A2* encodes a thiamine transporter protein. *SLC19A3* shares 39% homology with *SLC19A1* and 48% homology with *SLC19A2*. It is a new heterotrimeric carrier family along with *SLC19A1* and *SLC19A2* transporter proteins, which belongs to the second thiamine transporter and is a potential biotin transporter [56–58]. When extracellular thiamine concentrations are below 2 mmol/L, active transport via THTR2 is possible. However, at higher extracellular concentrations compared to intracellular concentrations, thiamine enters the cell by passive diffusion, so that exogenous thiamin supplementation remains effective even when *SLC19A3* is dysfunctional. In mammals, biotin serves as a coenzyme for several key carboxylases and also regulates several gene expression in vivo. Early biotin deficiency inhibits *SLC19A3* expression, but as the deficiency worsens, it instead stimulates *SLC19A3* expression and increases biotin transport [58].

Homozygous and mutated *SLC19A3* gene can cause various conditions such as biotin-thiamine responsive basal ganglia disease (BTBGD), lactic acidosis combined with encephalopathy, infantile epileptic spasms, and early childhood encephalopathy induced by disease or trauma. Among them, mutations such as p.Lys44Glu and p.Glu320Gln in the *SLC19A3* gene can manifest as WerNicke-like encephalopathy [59–61].

Biotin-thiamine-responsive basal ganglia disease, first described in 1998 by Ozand et al., is an autosomal recessive disorder that is most commonly observed in the Saudi Arabian population. Most parents are consanguineous and more than 100 cases have been reported [56, 60]. BTBGD usually occurs in children of preschool or school age and may be precipitated by febrile illness or minor trauma. Clinical symptoms include impaired consciousness, seizures, autism spectrum disorder and depression, as well as extrapyramidal and pyramidal fasciculus symptoms. The patient's intelligence is usually unaffected, and in severe cases mild mental retardation may remain. Typical seizure types are simple partial or generalized seizures that can be controlled with one antiseizure medication [62, 63]. Extrapyramidal symptoms may manifest as dystonia, dysarthria, and ataxia. Damage to the cone bundle can result in supranuclear facial nerve palsy, extraocular muscle palsy, hemiparesis, and so on [63–65]. Yamada et al. reported two cases of *SLC19A3* pathogenic homozygous mutations (C.958G>C, p.E320Q) with epileptic spasms but atypical EEG manifestations with multifocal spikes and no high-amplitude dysrhythmias [63]. MRI of the brain characteristically reveals centric necrosis of the caudate nucleus head bilaterally and partial or complete necrosis of the shell nucleus in individuals with BTBGD. In addition, there may be supratentorial, infratentorial, and cortical vasogenic edema, with cerebellar, brainstem and thalamic involvement in about 1/3 of patients [66, 67]. Gliosis and cerebral atrophy may occur in patients receiving untimely treatment [60]. In one case, increased T2 signal intensity was observed in the cervical segment of the spine [67]. In general, when encountering neurodegenerative disease, epilepsy, or dystonia, together with neuroimaging showing basal ganglia changes should be considered as a possibility of BTBGD [68].

In 2017, China Xuanwu Hospital proposed the following diagnostic criteria for BTBGD [69]: (i) subacute onset in early childhood; (ii) presence of seizures; (iii) manifestation of dystonia; (iv) display of ataxia; (v) presence of similar symptoms in other family members or children’s parents are consanguineous; (vi) identification of abnormal signals in the head and shell nuclei of the bilateral caudate nuclei visible on head MRI; (vii) absence of a better explaination by other diseases; (viii) genetic examination confirming the homozygous or heterozygous mutation of the *SLC19A3* gene. The diagnosis of BTBGD should be highly suspected if the patient meets any two of criteria (i) to (v) and fulfills criteria (vi) and (vii). The diagnosis of BTBGD can be confirmed when the patient's genetic testing meets criteria (viii).

Early treatment is particularly important for patients with BTBGD. Administering high doses of biotin (2–3 mg/kg/d) and thiamine (100–300 mg/d) shortly after symptoms onset can lead to complete resolution or remission of symptoms within a few days in the majority of patients. Failure to receive prompt treatment may result in sequelae such as dystonia, quadriplegia, and mental retardation, and even death. In some patients, using biotin alone may trigger a recurrence of the acute crisis, so biotin is usually combined with thiamine [67]. Once significant remission is achieved through acute treatment, a low-dose maintenance regimen is necessary for long-term management. However, *SLC19A3*-associated infantile epileptic spasms are ineffective with large doses of biotin, with only a transient response to ACTH.

### Biotin metabolism disorders and epilepsy

Biotinase deficiency is caused by mutations in the gene encoding biotinase (*BTD* gene), with 51% of cases attributed to the homozygous c.98-104del7ins3 mutation, It is an autosomal invisible genetic disorder with an estimated prevalence of about 1 in 60,000, and 20% of patients have a history of parental consanguinity [70]. Mutations in the *BTD* gene decrease biotin activity and reduce the ability of the intestine to absorb biotin, resulting in biotin deficiency. In mammals, biotin is a coenzyme of four major carboxylases (pyruvate carboxylase, propionyl coenzyme A carboxylase, 3-methylcrotonyl coenzyme A carboxylase, and acetyl coenzyme A carboxylase). Dysfunction of these four carboxylases can affect gluconeogenesis, lipid synthesis, and amino acid catabolism, leading to multi-system involvement in the body [70, 71].

Most symptomatic children with biotinase deficiency exhibit serum biotinase activity at 10% of normal levels or undetectable, and biotin levels in the brain and CSF are typically even lower [72]. The main manifestations of biotin deficiency are epilepsy, psychomotor retardation, deafness, abnormal muscle tone, skin manifestations, and decreased vision, which usually occur in the first few months of life. Epilepsy occurs in 55% of patients with the homozygous c.98-104del7ins3 mutation in the *BTD* gene, and for 38% of these patients, epilepsy is the initial symptom [73]. The most common types of seizures observed are generalized tonic-clonic, followed by myoclonus, epileptic spasms and Ohtahara syndrome [72, 74, 75]. Skin manifestations include rashes, skin infections, and hair loss, with rashes usually occurring on the face, eyes, mouth, and nose, and sometimes peeling skin [75]. These skin symptoms may disappear after 1–2 weeks of biotin treatment [76, 77]. Metabolic abnormalities affecting the respiratory center of the medulla oblongata may result in hyperventilation, apnea and laryngeal tinnitus. Some patients with biotinase deficiency may gradually develop ataxia with age.

The EEG findings in individuals with biotinase deficiency commonly reveal burst suppression and multifocal spikes [72]. Imaging demonstrates various manifestations, including diffuse abnormalities in the supratentorial white matter, reduced brain volume, diffuse cerebral edema, basal ganglia calcifications, and delayed myelination [78, 79]. Rarely, bilateral subcortical cysts in the frontal lobe, cranial thickening, and Dandy Walker variant may be observed [73]. MRI scans of three Chinese patients showed the lesions that could involve the spinal cord, with edema and progressive demyelination of the cervical and thoracic spinal cord [80].

Seizures are controlled by antiseizure medications in about half of patients and disappear after biotin administration, while in patients who do not receive antiseizure medications, most seizures stop within 24 hours of starting biotin application. Some patients with multiple recurrent metabolic disorders may develop irreversible neurological damage with residual mental retardation, ataxia, spastic paraplegia, sensorineural hearing loss, optic nerve atrophy, and epilepsy [79, 81]. Because valproic acid impairs mitochondrial function in the liver and reduces biotinidase activity, it should be avoided in this group of patients [82]. MRI, metabolic screening, and genetic testing should be performed as soon as possible in patients with infantile seizures to establish an early diagnosis and prevent neurologic sequelae with timely and effective treatment [79].

### Vitamin B12 metabolism disorders and epilepsy

Vitamin B12 deficiency is a common disorder that usually results in megaloblastic anemia, tongue inflammation, and neuropsychiatric symptoms [83]. The most common neurological manifestation is subacute combined degeneration of the spinal cord and peripheral sensory neuropathy, while epilepsy is a rare manifestation in cases of vitamin B12 deficiency [84]. Gramer et al. studied a cohort of 176,702 German children and found 33 children suffered from vitamin B12 deficiency with a prevalence of approximately 1:5355 [85]. Irevall et al. screened 35 cases of vitamin B12 deficiency in 11,143 infants in Sweden, with a prevalence of approximately 314 per 100,000 [86]. Variations in prevalence can be attributed to regional differences and dietary habits taken into account.

**Fig. 3:**
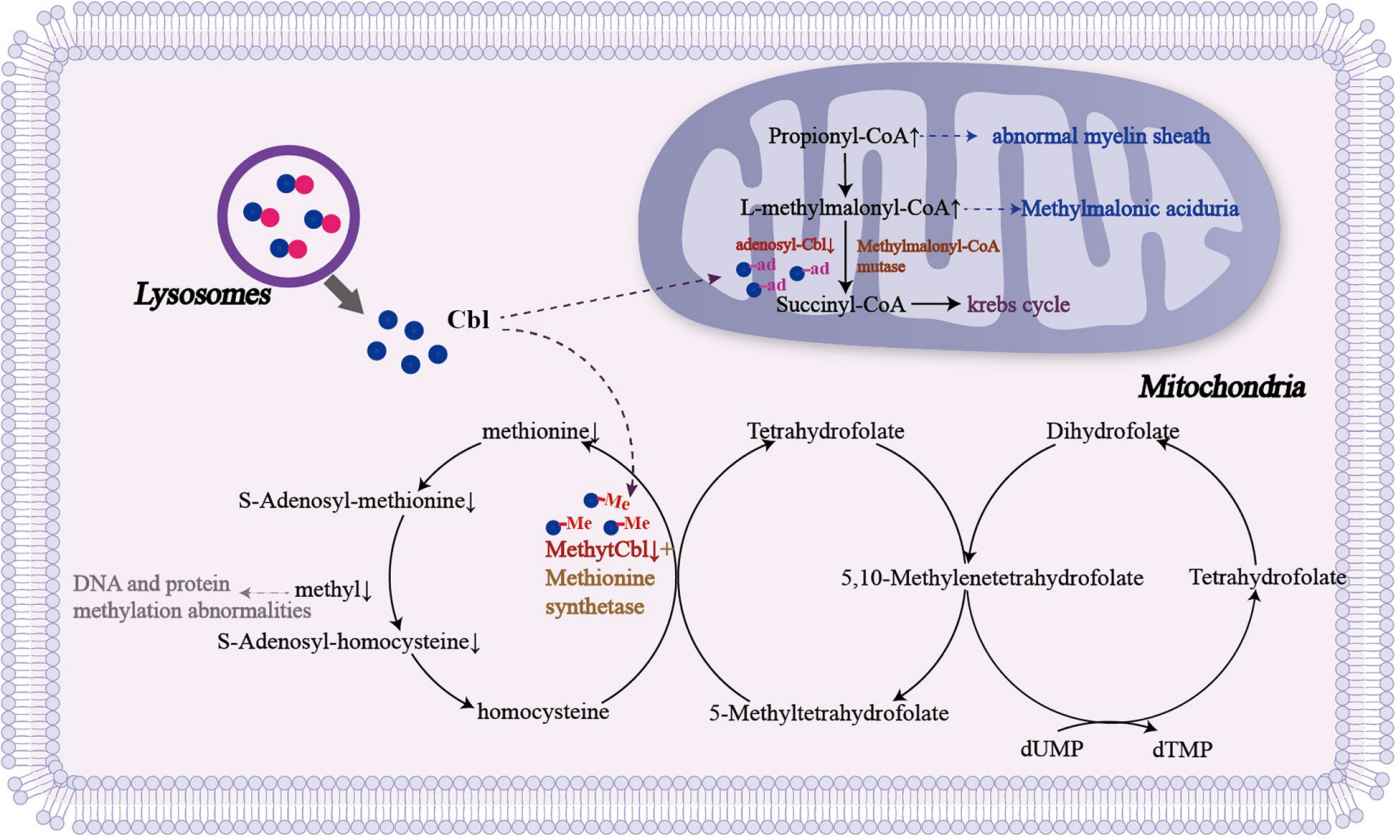
Metabolic pathways involving vitamin B12. Adenosylcobalamin is involved in the conversion of methylmalonyl coenzyme A to succinyl coenzyme A. In vitamin B12 deficiency, methylmalonyl coenzyme A and malonyl coenzyme A accumulate, leading to the synthesis of single-chain fatty acids, which are incorporated into the myelin sheath to cause myelin changes; homocysteine is converted to methionine in the presence of methylcobalamin, methionine is metabolized to S-adenosylmethionine, and S-adenosylmethionine (SAM) is demethylated and converted to S-adenosine homocysteine (SAH). The SAM : SAH ratio decreases in methylcobalamin deficiency, protein, lipid and neurotransmitter synthesis in the CNS is impaired, and DNA synthesis and cell division are inhibited

There are currently four theories about the mechanism by which vitamin B12 deficiency causes neurological disorders [87–89]: (i) vitamin B12 exists in two active forms, methylcobalamin and adenosylcobalamin. Adenosylcobalamin plays a role in converting of methylmalonyl coenzyme A to succinyl coenzyme A. In vitamin B12 deficiency, the accumulation of methylmalonyl coenzyme A and malonyl coenzyme A, leads to the synthesis of single-chain fatty acids that are incorporated into the nerve sheath, resulting in myelin changes; (ii) homocysteine is converted to methionine in the presence of methylcobalamin, which can be metabolized to S-methionine. However, in methylcobalamin deficiency, the conversion of methionine to S-adenosylhomocysteine (SAH) occurs after the formation of S-adenosylmethionine (SAM), leading to a decrease in the SAM : SAH ratio; (iii) homocysteine accumulation also stimulates N-methyl-D-aspartate (NMDA) receptors, leading to the development of neurodegenerative diseases; (iv) elevated levels of TNF-α have also been reported in patients with vitamin B12 deficiency, potentially impairing brain development (Fig. 3).

According to current case reports, the phenotype of epilepsy associated with noninfantile vitamin B12 deficiency remains uncertain and may present with complex partial seizures, generalized seizures, tonic clonus and generalized tonic-clonic convulsions. Additionally, patients may experience secondary systemic symptoms such as confusion, tendon reflex hyperactivity, reversible dementia, mood swings, psychotic symptoms, and hypertension,. Patients with epilepsy and other neuropsychiatric symptoms who receive cobalamin replacement therapy may resolve or even completely recover [84, 90–93]. However, in one case, psychotic symptoms and seizures recurred despite continuous cyanocobalamin replacement therapy after the discontinuation of antipsychotic and antiseizure medications [90].

Patients with infantile vitamin B12 deficiency commonly have vegetarian and pernicious anemic mothers who are also deficient in maternal vitamin B12. These patients usually show developmental delay before the age of 6 months, followed by the onset of seizures. It is worth noting that all reported cases of vitamin B12-deficient epilepsy in infancy and early childhood present as West syndrome [94–96]. Meena et al. found that children with infantile spasms had lower mean serum vitamin B12 levels compared to children with generalized developmental delay but without spasms. What’s more, children with infantile spasms had higher levels of serum homocysteine and urinary methylmalonic acid compared to the control group, suggesting a potential association between vitamin B12 deficiency and the occurrence of infantile spasms [97]. In addition to epilepsy, generalized developmental delay, coma, tachycardia, generalized hypotonia, profound hypotonia, microcephaly, active tendon reflexes, and loss of tendon reflexes may be present. A case reported by Pavone et al. described an infant who developed infantile spasms at 8 months of age, as evidenced by high amplitude dysrhythmia on the EEG. Treatment with a combination of vitamin B12, ACTH and vincristine improved neurological function within a few weeks, leading to the disappearance of spasms. However, the patient later developed disseminated bilateral myoclonic seizures with diffuse and widespread multi-spike activity on the EEG [96]. Glaser et al. reported a case involving a 6-month-old infant who was exclusively breastfed and with slowed background activity on the EEG. After starting vitamin B12 supplementation, the patient's cognitive development improved, but clustered spasms developed and the EEG showed hypsarrhythmia [93]. MRI results showed mainly reversible brain atrophy with delayed myelin formation, although some patients had normal MRI findings [94–96]. In some patients, MRI results may appear normal. Treatment combining with ACTH, antiseizure medications, and vitamin B12 in these patients has shown improvments in EEG features and imaging manifestations, leading to a reduction in seizures. However, there has been a reported case of clinical deterioration with the onset of infantile spasms despite near-normal levels of vitamin B12 in a patient receiving supplementation. Subsequent treatment with topiramate resulted in no further seizures. It suggests that the development of West syndrome in this patient may not be directly related to vitamin B12 deficiency [96]. In addition, metabolic analysis may show significantly lower serum levels of vitamin B12 and significantly higher levels of methylmalonic acid and homocysteine. Whereas the severity of vitamin B12 deficiency does not necessarily correlate with prognosis. Attention should be focused on indicators such as methionine and S-adenosylmethionine in plasma and methyl tetrahydrofolate in CSF [93, 95].

Overall, vitamin B12 deficiency is treatable and can lead to serious neurological sequelae if left untreated, so it is important to consider vitamin B12 deficiency as a potential cause in patients with unexplained epilepsy, especially when accompanied by macrocytic anemia [85]. Long-term use of antiseizure medications is generally discouraged, as medications such as carbamazepine and phenytoin can reduce vitamin B12 and folic acid levels. Hence, it is advisable to avoided these medications [98].

### Folic acid metabolism disorders and epilepsy

Folic acid is a coenzyme of the one-carbon unit transferase family of biochemical reactions in the human body and is involved in the conversion of homocysteine to methionine and the formation of the active methyl donor, SAM. SAM, in turn, provides methyl for the synthesis of DNA, fatty acids, phospholipids and proteins, with methylation of myelin proteins maintaining the stability of the myelin sheath. The conversion of homocysteine to methionine requires the availability of choline or folic acid to provide methyl groups, so when folic acid is deficient, cells metabolize more choline, resulting in a deficiency of phosphatidylcholine, a key component of mycelin. This can lead to a reduction in the stability of the myelin sheath [99, 100].

In 2004, Ramaekers et al. defined primary cerebral folate deficiency as a neurological syndrome associated with normal levels of extraneurological folate but reduced levels of 5-methyltetrahydrofolate (5MTHF), a folate metabolite in cerebrospinal fluid [101]. It may be due to impaired transport of folate into the brain or increased folate metabolism within the brain. Thus, the etiology of primary cerebral folate deficiency includes the presence of autoantibodies against the folate receptor (FR1) and mutations in the *FOLR1* gene, which encodes the FR1 protein [101]. Secondary cerebral folate deficiency encompasses conditions such as dihydrofolate reductase deficiency, genetic folate malabsorption, and dihydropteridine reductase deficiency.

#### Primary cerebral folate deficiency

Cerebral folate deficiency is a rare autosomal recessive disorder caused by mutations in the *FOLR1* gene,. To date, 19 cases of cerebral folate deficiency due to *FOLR1* gene mutations have been reported. The *FOLR1* gene encodes an adult folate binding protein, which is primarily expressed in the choroid plexus. This protein plays a crucial role in transporting tetrahydrofolate (FH4) across the blood-brain barrier. Patients with cerebral folate deficiency usually present with neurological manifestations, mainly ataxia, dystonia, psychomotor hypotonia and seizures after the age of 1 year. Other rare symptoms may include irritability, microcephaly, autism spectrum disorder, tremor, global developmental delay, coma, polyneuropathy, axial hypotonia, and daytime sleepiness [100, 102–108]. Among the seizures, myoclonic and GTCS are the most common, although some patients may experience drop seizures and epileptic spasms. It is important to note that patients in this group usually exhibit normal cognitive function. Brain MRI reveals several abnormalities in patients with cerebral folate deficiency, including delayed myelin development, cerebellar atrophy, cortical laminar necrosis, bilateral intrabasal ganglia calcifications, frontal lobe and cerebellar atrophy. Nuclear magnetic resonance spectroscopy analysis shows reduced peaks of choline and inositol. The impaired function of folate receptor protein 1 (FR1) function due to mutations in the *FOLR1* gene is responsible for these abnormalities. Furthermore, the levels of 5MTHF in the CSF are extremely low or even unmeasurable in this group of patients.

Another important cause of folate deficiency is the presence of autoantibodies against folate receptors in the patient's body, which block the transport of folate across the blood-brain barrier. It typically presents with restlessness, irritability, sleep disturbances initially, which gradually progress to psychomotor retardation, seizures, and cerebellar ataxia [101, 109]. The disease begins with restlessness, anger, and sleep disturbances. Some cases also present with coma, visual impairment, progressive acoustic hearing loss and spastic paraplegia [101, 109]. Similar to the *FOLR1* gene mutations, the most common seizure types observed in this condition are myoclonic and GTCS [101]. Neuroimaging studies may show frontotemporal atrophy, progressive supratentorial and infratentorial atrophy, cerebellar atrophy, and periventricular demyelination [101]. Magnetic resonance spectroscopy analysis often shows decreased levels of N-acetylaspartate [109]. In addition, the concentration of 5MTHF in the CSF is lower than normal in patients with idiopathic cerebral folate deficiency, and some patients also have reduced concentrations of CSF 5-hydroxyindoleacetic acid and biopterin.

Early initiation of folic acid supplementation (2–10 mg/kg/day) is recommended, and patients who begin supplementation before the age of 6 years tend to have a better prognosis than those who start supplementation after the age of 6 years [101]. Even if folic acid supplementation is commenced in adulthood, it can still help reduce the frequency of seizures and improve the patient's quality of life. The combination of oral folic acid with intravenous administration is considered more effective than oral administration alone [110]. However, there are cases where patients experience ongoing deterioration in their developmental status, even after initiating treatment with folic acid and various antiseizure medications at the age of 1 year, with seizures reaching more than 30 times per day [110]. In addition, the ketogenic diet has a better response when used as an additional treatment along with antiseizure medications [111]. CSF folic acid concentration should be monitored during folic acid supplementation therapy to prevent deficiency or overdose.

#### Secondary folic acid deficiency

The molecular basis of the inherited folate absorption disorder is a mutation in the *SLC46A1* gene, which encodes the proton-coupled folate transporter (PCFT). This transporter is responsible for absorption of folate in the intestine [112, 113]. In addition, patients with hereditary folate absorption disorders also have a folate transporter disorder in the central nervous system, suggesting that PCFT is also involved in the transport of folate to the brain [112]. Hereditary cerebral folate malabsorption is a secondary cause of cerebral folate malabsorption, with nearly half of the patients having a consanguineous parent and a mortality rate of 40%, with women being more susceptible than men [114]. The condition primarily manifests as megaloblastic anemia with recurrent diarrhea, mouth ulcers, recurrent infections, anemia and loss of appetite, which typically occur in the first months of life. Neurological manifestations include developmental delay, psychomotor retardation, behavioral abnormalities, ataxia, peripheral neuropathy, and seizures. Psychiatric symptoms such as insomnia, forgetfulness, irritability, depression, and schizophrenia may also occur [114]. These patients typically have low levels of folate in both plasma and CSF, and they may exhibit abnormally high urinary metformin glutamate excretion [115]. MRI scan may show calcifications in the basal ganglia region, occipital cortex, and internal capsule sites. For patients with impaired folic acid absorption, intramuscular folic acid injection may be considered as an alternative to bypass gastrointestinal absorption. If patients continue to have seizures despite folic acid supplementation, additional antiseizure medicatoins should be considered.

The *DHFR* gene is located at 5q14.1 and encodes dihydrofolate reductase. Dihydrofolate reductase is a key enzyme in folate metabolism in vivo, responsible for the reduction of dihydrofolate to tetrahydrofolate, which is involved in the methyl shuttle required for the ab initio synthesis of thymidylate and certain amino acids. Additionally, it serves as a cofactor for enzymes such as phenylalanine 4-hydroxylase, tyrosine 3-hydroxylase, and tryptophan 5-hydroxylase, which are required for the production of monoamines. Only six cases of dihydrofolate reductase deficiency have been reported to date, three of which were homozygous mutations in the *DHFR* gene p.Asp153Val and three were attributed to p.Leu80Phe mutations [116, 117]. The main symptom remains macrocytic anemia, which can be completely reversed by folic acid supplementation. Compared to patients with primary folate deficiency, those with dihydrofolate reductase deficiency typically exhibit less pronounced developmental abnormalities. However, they may experience respiratory infections, low intraocular pressure, microcephaly, ocular abnormalities, and seizures. The most common type of seizure observed is eyelid myoclonus with absence [117]. Additionally, strabismus and impaired vision may also be present [117]. CSF levels of 5-MTHF and tetrahydrobiopterin are lower than normal, while serum levels of folate, dihydrobiopterin, and homocysteine remain within the normal range. There is a slight decrease in the levels of monoamine metabolites such as homovanillic acid (the major catecholamine metabolite) and 5-hydroxyindoleacetic acid (5-hydroxytryptamine metabolite) [117]. MRI may show cerebral white matter atrophy, ventricular enlargement, periventricular gliosis, optic nerve atrophy, delayed myelin formation or dysplasia, cerebellar hypoplasia, cerebellar hemisphere atrophy, and hypersignal in the subcortical white matter [116, 117]. Folic acid supplementation can alleviate the symptoms of anemia in patients with dihydrofolate reductase deficiency, but this does not correct the deficiency of tetrahydrofolate in the brain, often leaving neurological sequelae. Furthermore, dihydrobiopterin plays a role in maintaining the reduced state of tetrahydrofolate, so the clinical phenotype of dihydrobiopterin deficiency is similar to that of dihydrofolate reductase deficiency. However, patients with dihydrobiopterin deficiency may develop early brain atrophy and intracranial perivascular calcifications [118, 119].

3-phosphoglycerate dehydrogenase (3-PGDH) deficiency is a rare autosomal recessive disorder of serine synthesis that has been reported in fewer than 20 patients. Deficiency of the 3-PGDH enzyme results in decreased serine synthesis in the body. Serine normally reacts with tetrahydrofolate to form glycine and methylenetetrahydrofolate, which is eventually reduced to 5-methyltetrahydrofolate [120]. Thus, impaired serine synthesis eventually leads to folic acid deficiency. Patients in this group usually present with microcephaly, severe psychomotor retardation, and spastic quadriplegia. Seizures usually occur within the first 2 years of life, and about half of the affected individuals present with West syndrome [121]. Previous reports have suggested that oral supplementation of serine can improve seizures. However, a study investigated the effects of L-serine supplementation in two patients with PGDH deficiency and found that it did not effectively control seizures. Interestingly, with glycine was added to the treatment regimen, seizures were completely controlled, and abnormal brain discharges in the patients showed improvement [120, 122].

## Conclusions

In conclusion, vitamin metabolism disorder-related epilepsy is a type of treatable metabolic epilepsy (Table 1). For patients with unexplained mental and motor developmental delay, seizures, recurrent acute encephalopathy, and systemic multisystem manifestations, it is important to conduct early diagnostic investigations such as cranial MRI, EEG, plasma amino acid metabolism, urine organic acid metabolism analysis, CSF metabolism, and genetic testing to clarify the diagnosis. Supplementation with pyridoxine, vitamin B12 and folic acid may be considered. It is advisable to avoid valproic acid, as it can impair liver mitochondrial function and cause hepatotoxic, while carbamazepine and phenytoin can decrease vitamin B12 and folic acid levels, therefore, these drugs should be avoided whenever possible. Levetiracetam and broad-spectrum antiseizure medications such as benzodiazepine may be chosen for treatment. In addition, early prevention is equally important, and individuals at risk should receive appropriate vitamin supplementation during pregnancy. It is expected that further research into the underlying metabolism mechanisms of epilepsy will lead to the development of more innovative treatment strategies, the discovery of more treatable types of epilepsy and greater therapeutic benefit.

**Table 1:** Clinical features of epilepsy associated with disorders of vitamin metabolism

Gene	Disease	Clinical manifestations	Metabolic abnormalities	Diagnostic	Treatment
*PLPBP*	Vitamin B6-responsive epilepsy	Severe ID, epilepsy, anemia, gastrointestinal dysfunction, necrotizing enterocolitis, gastroesophageal reflux disease, and metabolic acidosis.	Plasma: glycine↑ CSF: lactic acid↑, homovanillic acid↓,3-O-methyldopa↑	Standard vitamin B6 test; genetic testing	PN or PLP
*ALDH4A1*	Type II hyperprolinemia	Fever-related seizures, DD, ID, behavioral disorders, upper respiratory tract infections, diarrhea, abdominal pain, hyperlactatemia, hyperkalemia, and so forth.	Plasma: proline↑ Urine: urineP5C↑, proline↑, hydroxyproline↑, glycine↑	Genetic testing	PN or PLP
*ALDH7A1*	Pyridoxal-dependent epilepsy	Intrauterine fetal distress, epilepsy, mental disorders, DD, endocrine metabolic disorders.	Plasma: serine↑, glycine↑, taurine↑, histidine↑, 3-methoxytyrosine↑ CSF: GABA↓, glutamate, serine↓, glycine↓, taurine, histidine↓, 3-methoxytyrosine↓ Urine: α-AASA/creatinine↑, 2-OPP↑	Urinary α-AASA/creatinine and 2-OPPdetection; Genetic testing	PN or PLP; arginine; restrict lysine intake
*PNPO*	Pyridoxamine 5'-phosphate oxidase deficiency	Prematurity, epilepsy, DD, ID, anemia, coagulation disorders, gastrointestinal manifestations.	CSF: glycine↑, PM↑, PLP↓ Urine: vanillic acid↑ Plasma: PM↑	Measurement of CSF and plasma PM levels; Dry blood spot assay for PNPO enzyme activity; Genetic testing	PLP (44% of patients responded to PN)
*TNSALP*	Hypophosphatemia	Epilepsy, calcium-phosphorus metabolism abnormalities.	Plasma: PLP↑, phosphoethanolamine↑ Urine: phosphoethanolamine↑	Plasma PLP assay	Enzyme replacement therapy; bone marrow and stem cell transplantation
*SLC19A3*	Biotin-thiamine responsive basal ganglia disease	Impaired consciousness, epilepsy, ADHD, as well as depression, extrapyramidal symptoms, and pyramidal tract symptoms; intelligence is usually not affected.		Genetic testing; Cranial MRI	Biotin and thiamine (after acute phase treatment relief, require low-dose maintenance in long-term therapy)
*BTD*	Biotinidase deficiency	Epilepsy, DD, deafness, abnormal muscle tone, skin manifestations, and visual impairment.	Metabolic acidosis	Genetic testing	Biotinidase
*/*	Vitamin B12 deficiency	Epilepsy, impaired consciousness, hyperreflexia, reversible dementia, mood swings, psychiatric symptoms, hypertension.	Plasma: Vitamin B12↓, methylmalonic acid↑, homocysteine↑	Plasma Vitamin B12 assay	Vitamin B12 (avoid using carbamazepine, phenytoin)
*FOLR1*	Primary cerebral folate deficiency	Cerebellar ataxia, muscle tone disorders, reduced psychomotor function, and epilepsy; less common symptoms include irritability, microcephaly, autism spectrum disorders, tremors, global developmental delay, coma, multiple neuropathies, axial low eye pressure, and daytime sleepiness.	CSF: 5-methyltetrahydrofolate↓	MRS; genetic testing	Folic acid
*SLC46A1*	Secondary folic acid deficiency	DD, ID, cerebellar ataxia, peripheral neuropathy, and epilepsy; insomnia, forgetfulness, and psychiatric symptoms may also occur; macrocytic anemia, recurrent diarrhea, oral ulcers, recurrent infections, anemia, and loss of appetite.	Plasma and CSF: folic acid↓ Urine: N-methylglutamine↑	Plasma folic acid assay; Genetic testing	Folic acid (intramuscular injection)
*DHFR*	Secondary folic acid deficiency	Mild DD, microcephaly, ocular abnormalities, epilepsy, respiratory tract infections, low eye pressure, macrocytic anemia.	CSF: 5-MTHF↓, tetrahydrobiopterin↓, vanillic acid↓, 5-hydroxyindoleacetic acid↓	Genetic testing	Folic acid
*PHGDH*	3-phosphoglycerate dehydrogenase deficiency	Microcephaly, severe psychomotor impairment, spastic quadriplegia, epilepsy.	Plasma: serine↓, folic acid↓, glycine↑	Genetic testing	Serine and glycine

*Abbreviation*: *PN* Pyridoxine, *PLP* Pyridoxal 5'-phosphate, *P5C* Pyrroline-5-carboxylate, *α-AASA* α-aminoadipic semialdehyde, *2-OPP* 6R-oxopropylpiperidine-2-carboxylic acid, *PNPO* Pyridoxal (amine) 5'-phosphate oxidase, *ID* Intellectual disability, *DD* Developmental delay, *ADHD* Attention deficit hyperactivity Disorder, *CSF* Cerebrospinal fluid, *MRI* Magnetic resonance imaging, *MRS* Magnetic resonance spectroscopy

## Data Availability

Not applicable.

## Abbreviations

2-OPP: 6R-oxopropylpiperidine-2-carboxylic acid
3-PGDH: 3-phosphoglycerate dehydrogenase
5-MTHF: 5-methyltetrahydrofolate
AADC: Aromatic L-amino acid decarboxylase
ADHD: Attention-deficit/hyperactivity disorder
ALDH4A1: Acetaldehyde dehydrogenase 4 family member A1
ALDH7A1: Acetaldehyde dehydrogenase 7 family member A1
BTBGD: Biotin-thiamine responsive basal ganglia disease
BTD: Biotinase deficiency
FH4: Tetrahydrofolate
FR1: Folate receptor protein 1
GSA: Glutamate semialdehyde
GTC: Generalized tonic-clonic
GTCS: Generalized tonic-clonic seizures
HPII: Type II hyperprolinemia
HVA: Homovanillic acid
NMDA: N-methyl-D-aspartate
P5C: Pyrroline-5-carboxylate
P5CDH: Delta(1)-pyrroline-5-carboxylate dehydrogenase
P6C: Piperideine 6-carboxylate
PCFT: Proton-coupled folate transporter
PL: Pyridoxal
PLP: Pyridoxal 5'-phosphate
PLPHP: Pyridoxal phosphate homeostasis protein
PM: Pyridoxamine
PN: Pyridoxine
PNPO: Pyridoxal (amine) 5'-phosphate oxidase
PPI: Inorganic pyrophosphate
SAH: S-adenosylhomocysteine
SAM: S-adenosylmethionine
SIA: Seizures with impaired awareness
THTR2: Thiamine transporter protein 2
TNSALP: Tissue non-specific alkaline phosphatase
α-AASA: α-aminoadipic semialdehyde
